# The Texas State Medical Association, Section on Obstetrics and Diseases of Children

**Published:** 1900-10

**Authors:** J. J. Robert

**Affiliations:** Hillsboro, Texas


For Texas Medical Journal.

### THE TEXAS STATE MEDICAL ASSOCIATION, SECTION ON OBSTETRICS AND DISEASES OF CHILDREN.

### Some Requisites for Successful Work in this Department.*

BY J. J. ROBERT, M. D., HILLSBORO, TEXAS.

Custom demands that the chairman of this section give some brief résumé of the scientific advancements, in this branch of our profession, during the past twelve months. To prepare some fitting paper along that line was originally my intention, but the numerous cares of a busy practice have from time to time made such demands upon me that I have found myself unable to do the subject full justice. Then again, so far as the practice of obstetrics is concerned, in the work of the general practitioner there have been very few advancements made. The same old science of accouchment holds good, that, when we were students, seemed to worry our minds so much. The precepts of the olden masters are still good in practice, and by reference to the text they will tell you far more pleasantly and instructively than I could hope to do, those things calculated to serve you best in times of need.

With the assistance of my co-laborer, the secretary of this section, Dr. Burroughs, I have tried to provide you a good program. That our efforts have not been more successful we both regret very much, and yet the reply to many of our cards came back that the gentlemen

*Chairman's Address, Section on Obstetrics and Diseases of Children, read before the Texas State Medical Association at Waco, July, 1900.

were pledged to assist other sections. Indeed, I have almost been forced to believe that the opinion obtains very extensively, that any old woman can attend to a case of obstetrics, and almost anybody can nurse a poor little sick child back to health. Very often can I remember hearing it sneeringly said in regard to the physician, "Oh, he is a pretty fair granny, or he is a very good doctor for children," just as if it required no skill nor study in either case. Now this should not be so. For in all the realms of scientific medicine, I can imagine no field more crowded with opportunities for doing good work, than the sphere covered by this section. No man who has watched a suffering woman in the fearful ordeal of childbirth can, if he be a man, regard the same in any but a serious light. The highest office conferred upon womankind is that of giving birth to sons and daughters and the next in importance is the careful and proper development of these helpless beings, rearing them to a glorious youth, and safely piloting them along the many rough places during the earlier years of their childhood. The royal diadem of the most kingly king known upon earth in its intrinsic value is to my mind but poor dross when compared with emotions, of manly and professional pride and gratification experienced by me so often in life, when I had administered some small assistance to a good woman in protracted labor. So also all the wealth of the Rothchilds could never purchase the joy that I have felt, away down deep in my heart, after days and nights of anxious, careful watching, a devoted parent is made to rejoice, and sunshine has again come to some happy home because the dear little invalid who has been so very near to death's door, through my instrumentality, by divine direction, had once again put on the smile and playful air of returning health. The value of these pleasures is fully known to every physician of any experience.

To my mind, the importance of the duties of the obstetrician is very much underrated. His office is not simply to receive the child and tie the cord, almost any old woman might do that, but to the doctor who would do his duty correctly, and best protect the life and health of his patient, his responsibility begins in early pregnancy, and continues to its termination. Experience has taught me how difficult it is to impress the laity with the necessity for such care. Indeed, it is lamentably true that the average man would give more considerate care to the fine mare or the jersey heifer than to the woman who is to mold the life and character of his own offspring. And if perchance the doctor does his duty, he is too often charged with simply a disposition to multiply his visits for mercenary gain. I make it my rule, at least, to try to do my duty as I see it, regardless of the opinion of others. If the pregnant woman is in a family in my regular practice, I watch her carefully from time to time during the entire period of gestation, in order to prepare her in every way possible for passing safely through the troubles ahead of her. If the party be a stranger to me, and I am, during the latter weeks of gestation, engaged to attend her, I make it my rule to at once call upon the woman for the purpose of mutual acquaintance between patient and physician. This I think is always proper, first, because the woman will very naturally feel more at ease when labor comes on, if her attendant is not an entire stranger to her. And in the second place for the more important reason that I am anxious, for her own safety, to know the exact condition of her general health. Now, in all the years of my practice, while I generally interrogate my patient very closely at such a visit, I can recall very few instances in which it was necessary to subject her to other than an ordinary conversational examination. The condition of the bowels, the action of the kidneys, the history of former confinements, if there have been such, the action of the heart, the request for a sample of urine for analysis, the general directions for care of the breasts in the primipara, are points of interest that may be inquired into and advised upon easily by the expert obstetrician with very little embarrassment to the woman, and the very fact that her attendant is instructed along these lines, not only puts him in possession of much useful knowledge, for future guide, but the care thus manifested in his patient at once, and I think most legitimately, obtains for him the perfect confidence of the woman whose life and future health is confided into his keeping. It does seem that implicit confidence upon the part of a woman, in her attendant, is absolutely needed, and I think that nothing honorable should be left undone to produce this feeling of ease and confidence. Many instances which still crowd my memory have taught me that a positive knowledge of the true condition of my patient enables me, by a cheerful, confident demeanor and by *judicious* expressions, to administer not only the needed assistance at the proper time, but also much courage and contentment to her mind. Furthermore I venture the assertion with no fear of contradiction that the obstetrician never does better work than when he knows, beyond a doubt, that he enjoys the full confidence of the suffering woman. One instance I recall which has left such an impression upon my life, that I doubt very much if eternity itself can efface it. Simple and yet pleasingly impressive, it occurred thus: a lovely young woman, in one of the most devoted families ever entrusted to my care, had already experienced one or two accidents in the earlier weeks of pregnancy. During all of this gestation I had watched her with more than ordinary care. All the solicitude that good women experience during these months, seemed to have been intensified many fold with her. The confidence placed in me by herself and her family, seemed almost supernatural. Many of the older of you gentlemen can doubtless recall similar emotions when I assure you that I sometimes was weighed down by this manifestation of confidence, fearful lest I should prove unworthy. So when her labor began and I had made my examination, the woman being quite small and very compactly built, you can imagine my disappointment upon discovering a breach presentation. Disappointed, because I feared for the safety of the child. Now I had promised this patient that I would let her know her exact condition under all circumstances, so I explained to her the presentation, the danger to her child, and the tedious character of labor she might anticipate. God grant that I may never forget the complacent look of confidence manifested in her countenance, when she said to me, "It is all right, doctor, I am in your hands, and I know you will help me all that any one could do, and I am sure that you will save my child." Almost without my own knowledge I assisted the dear little woman, and she helped herself, and in all my experience I can recall no case of labor which, *under such circumstances,* was accomplished more easily and naturally than this. The sunlight which entered that home then has never lost its brightness, and as for myself, why the pen of the poet or the artist's brush hath never attained such perfection as to enable them to describe the influence upon my life for good which has almost daily been exerted by that babe, now grown to be a sweet little girl of five summers, as she runs to meet me, no matter where she meets me, with outstretched arms and smiling lips, making me so thankful that I assisted her into life. Surely, you will pardon the recounting of so simple an occurrence, in which I only did my duty as any doctor should do, but it impresses the point I desire to make, the pleasant satisfaction and real helpfulness to the physician of such confidence. And yet the honorable obstetrician should not and would not prove recreant to this trust, either by doubtful methods or reckless procedure.

Again I suggest that the services of obstetricians are not over when labor is finished. Daily I become more convinced by actual experience that the doctor who assumes to attend a woman in her confinement should, if at all practicable, see her once a day for several days after labor. It is the custom in this State, at least in the country and smaller towns, to see the patient very rarely more than once either before or after labor. This is not right, and it is due very largely to the fact that the doctors fail to educate their patients along this line. But when I begin to consider the duties of the general practitioner in this branch of his life's work, the cares and the responsibilities multiply so rapidly, in my mind, that I scarcely know where to begin nor where to finish. A "meddlesome midwifery" should not be encouraged, and yet to sit idly by and wait for "nature to take its course" often results in very much unnecessary suffering and delay. The knowing what to do and how, and when to do, is the very important lesson to be acquired.

The experience of the past has taught us in no uncertain manner that there are circumstances under which no physician should allow himself to attend a woman in confinement. To the younger gentlemen here present, I address this reminder. The intimate and unmistakably demonstrated relationship between scarlet fever and erysipelas and the much dreaded puerperal fever is impressed upon our minds in the texts, and in the lecture room, but it remains for those of you who have no real experience along this line to yet see in full power the sad practical demonstration of the truth that no honest or truthful physician can afford to go to a case of labor while treating either erysipelas or scarlet fever. Every doctor knows this, and yet it is surprising how few of the laity ever dream of such a thing. Antiseptic precautions, scrupulously observed, have, I know, robbed this idea of many of its horrors, but the danger does exist, and the physician should allow no hope of reward, either financial or otherwise, to persuade him to fail in his duty by declining a case coming to him under such circumstances. A brief retrospect brings back to my mind the agony of disappointment in the early part of my professional life, when I yielded to the entreaties of a messenger who came for me, and went to attend a labor while I was treating a severe case of facial erysipelas. I had attempted to show him the danger of infection to the woman. By the free use of antiseptics I put myself in the best condition I possibly could, but the lurking little germ got in its work, and a critically ill woman was brought very near to death, and after weeks of suffering she thanked me for having saved her life when I realized that it was the lack of true courage upon my part that in all probability caused her to so suffer. That experience was dearly purchased by me, for no man except a physician who has gone through a similar case can imagine the heartaches that came to me during my attendance upon that one. I remember well how I tried to persuade myself that I was not to blame, but hourly the fear haunted me that I had not done my duty. It is true that the woman finally recovered from that illness, but the joy then experienced by me caused me to refuse positively, from that day to this, ever to go again to the puerperal room under such circumstances. These are the times when a doctor can prove himself a man worthy to be trusted for having assumed the rôle of attendant upon a woman in childbirth, he can, by honest, conscientious and skillful tact bring much comfort and sunlight into the home even during this trying ordeal. While the implicit confidence placed in her physician by the puerperal woman, as above referred to, is greatly to be desired, it is also needed that in return the physician, under any and all circumstances, should be true to his calling and scrupulously honest with his patient, guarding her from every accident with watchful eye and steady nerve. As for myself, I anticipate that no greater joy could attend me in my old age than to recall a life correctly devoted to the scientific practice of obstetrics. As the years go hurrying by, wealth and fame and professional honors may all be swept away, but the quiet satisfaction of having done your very best to alleviate the suffering of the good women that have been entrusted to your care will, I think, not only shed a halo around your declining age, but prove to you a welcome sesame to even far greater joys and more lasting rewards in the great hereafter.

The second division of this section, the Diseases of Children, is almost inseparable from the first, because in almost every family the physician who attends the mother is also the custodian of the child's health. Having already occupied so much of your time, I feel unwarranted in detaining you longer by any effort to refer to the numerous ailments of earlier infancy or childhood, and yet we all know that the quickest way into the hearts and home of the parents is by the faithful, tender and successful treatment of the little child. The dangers that beset the little one from the instant of its first wailings all along the years of childhood are so numerous and often so troublesome that the wisest heads are sorely puzzled and the stoutest hearts dismayed, because of the inability either to clearly diagnose or successfully treat the ailment. Statistics teach us that at least one-third of all our work is among children, and from the nature of their diseases and the tender susceptibility of their age one child in every five dies during the first year of life. The frailties of the body, the extreme and very delicate sympathies between different organs, the possibility even of a slight local disease extending to the entire system all render it often difficult to determine the true condition. Therefore, every ailment, however trivial it may seem to us, warrants a double measure of anxiety. One of our authors on the subject expresses it thus: "It is not mere hyperbole to say that you have to study a new semiology to learn a new pathology and new therapeutics, the old means of investigation will often fail you, and you will almost have to learn your alphabet again." This department of medicine requires more than ordinary skill and attention, for we are deprived of so many aids which older patients can afford us. It is often the case that the marked disturbances of pulse, temperature and respiration result from trivial causes. These manifestations convey to the physician a far different knowledge in the child and in the adult. Doubtless many of you can recall the hour when, sitting by some suffering little infant, with all your boasted skill and learning you felt utterly powerless, really unable to make a diagnosis in any way even satisfactory to your own mind. You were uncertain as to what was best to be done just then, and absolutely terrified by the thought of what might develop the next hour. It has been under such conditions that I have realized most forcibly the manifold responsibilities devolving upon the physician. The dawning of the new era is very near in which the most successful scientific devotee at this especial shrine will be the man who gives the least medicine under such circumstances. It is at this period that hygiene and proper nourishment are our most valued resources, and then it is that prophylactic measures should be carefully practiced. Start the child on its journey of life in the best way possible, and you will escape much worry, and the infant be saved much suffering which results from careless attention soon after birth. The filthy practice of just greasing the babe in rancid lard or goose grease and putting it aside for the first day or night, though still in use among certain classes, should never be allowed. I should insist, no matter how other babies have been handled, that the infant be thoroughly bathed, the umbilical stump dressed in good antiseptic fashion, with a soft, comfortably fitting band, wrap the little one in comfortable woolen and at once, as in olden times, put it to the mother's breast. Now, I know, it is a fad with some doctors to leave off the band and discard any dressing of the cord. This practice I have never adopted, and do not think I shall. And again, artificial feeding during the first few hours of infantile life is frequently the beginning of severe alimentary derangements. Nature's storehouse for the new-born babe is the mother's breast, and in the large majority of cases, if the proper care and attention has been given them during the latter weeks of gestation, in this emergency the supply will generally be equal to the demand.

I confess I am more devoted to this branch of my profession than any other. I love children. I delight to watch over and care for them. The unselfish confidence of a little child, whose fevered brow has been cooled, and whose faltering heart-throb, coaxed back to healthful vigor by some effort of mine, goes further toward leveling down the many rough places in my professional life than all the flattering encomiums of praise that the most potent magnate could bestow. In childhood's days our mothers sang to us the beautiful story of the greatest Physician the world hath ever known, taking a little child in His arms and proclaiming that of such the beautiful host in the kingdom of heaven is composed. So these pure and helpless little innocents are to us, as physicians, a double care, worthy the study and devotion of a lifetime.

In this rambling and hurriedly written paper I have refrained from any effort to offer you scientific thought. My only desire has been to present you the subject matter of this section, "Obstetrics and Diseases of Children," in such a manner that your interest therein might be aroused so that in subsequent years many of the members of this Association may feel inclined to contribute to this section their valuable assistance which we have so anxiously sought but failed to obtain.

And, gentlemen, I feel quite confident that if the faithful St. Peter should ever throw open wide the portals that guard the entrance to the New Jerusalem and allow any one of you to enter there, the sweetest songs of all the angels to your ears will be those of welcome which shall greet you, because of some kindly help and comfort that you have given either to some good woman in confinement or to the helpless little children to whom you have faithfully and tenderly administered.

